# Progress in Radiotherapy for Cholangiocarcinoma

**DOI:** 10.3389/fonc.2022.868034

**Published:** 2022-04-14

**Authors:** Ningyu Wang, Ai Huang, Bohua Kuang, Yu Xiao, Yong Xiao, Hong Ma

**Affiliations:** ^1^Cancer Center, Union Hospital, Tongji Medical College, Huazhong University of Science and Technology, Wuhan, China; ^2^Department of Gastrointestinal Surgery, Union Hospital, Tongji Medical College, Huazhong University of Science and Technology, Wuhan, China

**Keywords:** cholangiocarcinoma, indications for radiotherapy, target area delineation, radiotherapy dose, radiotherapy mode

## Abstract

Cholangiocarcinoma (CCA) originates from the epithelium of the bile duct and is highly malignant with a poor prognosis. Radical resection is the only treatment option to completely cure primary CCA. Due to the insidious onset of CCA, most patients are already in an advanced stage at the time of the initial diagnosis and may lose the chance of radical surgery. Radiotherapy is an important method of local treatment, which plays a crucial role in preoperative neoadjuvant therapy, postoperative adjuvant therapy, and palliative treatment of locally advanced lesions. However, there is still no unified and clear recommendation on the timing, delineating the range of target area, and the radiotherapy dose for CCA. This article reviews recent clinical studies on CCA, including the timing of radiotherapy, delineation of the target area, and dose of radiotherapy. Further, we summarize large fraction radiotherapy (stereotactic body radiotherapy [SBRT]; proton therapy) in CCA and the development of immunotherapy and the use of targeted drugs combined with radiotherapy.

## Introduction

Cholangiocarcinoma (CCA) is the second-largest hepatic malignancy after hepatocellular carcinoma (HCC), and recently, its incidence is increasing each year. The incidence of CCA varies geographically (1, 2), the incidence in Southeast Asian countries is much higher than that in Western countries. Currently, the reported incidence in China is approximately 6/100,000 people per year (3). According to the anatomical location of tumor occurrence, CCA can be divided into intrahepatic cholangiocarcinoma (ICCA) and extrahepatic cholangiocarcinoma (ECCA). ECCA can be further divided into perihilar cholangiocarcinoma (pCCA) and distal cholangiocarcinoma (dCCA) (4). CCA has a high degree of malignancy and poor prognosis. The 5-year survival rate of ECCA is 17%, while the 5-year survival rate of ICCA is only 5% (5).

Radical resection is the only cure for primary CCA (4). The method of surgical resection and the area of lymph node dissection depends on the site and the extent of tumor involvement (6). Since the clinical symptoms of CCA lack specificity, most patients would have reached the advanced stage of the disease at the time of diagnosis and lost the chance of radical surgery. Even at an early stage, the postoperative recurrence rates are high (7). Radiotherapy is an important means of local control, known as “the invisible scalpel”. In recent years, with the advancements in imaging and radiotherapy equipment and the progress of radiotherapy technology, the status of radiotherapy in the treatment of CCA has been greatly enhanced, and the importance of radiotherapy has also been clarified in recent studies. This article reviews recent clinical studies on CCA, including the timing of radiotherapy, delineation of the target area, and dose of radiotherapy. Further, we summarize large fraction radiotherapy [stereotactic body radiotherapy (SBRT); proton therapy] in CCA and the development of immunotherapy and the use of targeted drugs combined with radiotherapy.

## Radiotherapy for CCA

### Adjuvant Radiotherapy for Resectable CCA

CCA is highly malignant and progresses rapidly. Although surgery is the only treatment that can achieve a radical cure, radical resection is not advised for all patients, resulting in a 5-year survival rate of only approximately 30% after surgery (3, 4). As early as 2002, Nakeeb et al. conducted a retrospective study using the data of the 10-year postoperative follow-up of patients with ICCA or ECCA and found that the positive postoperative resection margin and lymph node metastasis were vital factors affecting the progression-free survival (PFS) time after surgery for CCA (8). At the same time, postoperative adjuvant chemoradiotherapy resulted in a significant survival benefit compared with surgery alone. Subsequently, Horgan et al. analyzed 20 studies on adjuvant therapy for CCA, and the meta-analysis found that adjuvant chemotherapy or adjuvant chemoradiotherapy had a better survival benefit than surgery alone for patients with positive lymph nodes and R1 resection (OR, 0.49; P ≤ 0.004 and OR, 0.36; P ≤ 0.002) (9). Since then, several retrospective studies or meta-analyses based on ICCA and ECCA have confirmed the crucial role of postoperative adjuvant concurrent chemoradiotherapy in prolonging survival and reducing the risk of death in patients with positive margins or positive lymph nodes (10–13). Therefore, existing guidelines and consensus recommend that postoperative adjuvant radiotherapy or chemoradiotherapy should be considered for ICCA and ECCA patients with positive margins or regional lymph nodes (14).

In addition, there is another landmark study in the history of postoperative adjuvant therapy for CCA, namely, the phase II clinical study, SWOG S0809 (15). To date, this is the only prospective study of adjuvant radiotherapy after CCA. A total of 79 patients with advanced CCA with a postoperative staging of pT2–4, N+ were enrolled in this study, and 25 of them underwent R1 resection. After surgery, all patients received postoperative adjuvant chemotherapy consisting of gemcitabine combined with capecitabine for 2–4 cycles, followed by capecitabine-based concurrent chemoradiotherapy. The dose of radiotherapy was 45 Gy in the lymphatic drainage area and increased to 54 Gy in the tumor bed. For R1 resection, the local dose could be increased to 59.4 Gy. The results showed that the median overall survival (OS) of postoperative radiotherapy for R0 was 34 months, which was higher than that reported in historical studies. Meanwhile, the median OS of R1 patients receiving radiotherapy after surgery was 35 months, and there was no difference in postoperative survival between R1 patients and R0 patients. These results suggest that postoperative radiotherapy and chemotherapy can not only effectively improve the survival of patients undergoing R1 resection but also bring survival benefits to patients in local advanced stages, such as T3 and 4.

Based on the above findings, the 2021 Chinese Society of Clinical Oncology (CSCO) guidelines suggest that postoperative adjuvant radiotherapy or chemoradiotherapy should be considered in ICCA and ECCA patients with positive margins or regional lymph nodes. Meanwhile, it is recommended that postoperative adjuvant radiotherapy should also be considered for patients with postoperative stage PT3–4 for ECCA, but this is not a priority recommendation (14).

### Neoadjuvant Radiotherapy for CCA

Orthotopic liver transplantation (OLT) is an optional treatment method for advanced ICCA, but previous studies have shown that liver transplantation alone could not provide a clear survival benefit. In 1993, the Mayo Clinic introduced neoadjuvant radiotherapy in 19 ICCA patients before OLT, which was followed by external irradiation of 45 Gy followed by intracavitary supplementation of 20–30 Gy, combined with 5-Fu sensitization, followed by 5-Fu chemotherapy until OLT. OLT was successfully implemented in 11 patients; the disease-free survival (DFS) at 3 years was 92% with a median follow-up of 44 months (16). Subsequently, a retrospective analysis of 37 patients with advanced ICCA undergoing OLT from 1985 to 2009 at the Mayo Clinic found that neoadjuvant chemotherapy or chemoradiotherapy resulted in significant survival benefits compared to postoperative adjuvant or surgery alone (5-year DFS: 47% vs. 33% vs. 20%, P=0.03) (17). At the same time, UCLA (the University of California, Los Angeles) Cancer Center conducted a follow-up analysis of the prognosis of ICCA patients undergoing OLT and found that in addition to tumor biological characteristics, lymph node metastasis, and distal metastasis were important factors affecting prognosis after OLT, and neoadjuvant therapy was also an independent factor affecting prognosis. Thus, neoadjuvant chemoradiotherapy is recommended for patients with high recurrence risks. For patients with a tumor length ≤ 6 cm, conventional radiotherapy or short-course large fraction radiotherapy (SBRT 40 Gy/5F) can be chosen based on the experience of the UCLA Cancer Center. While, For tumors larger than 6 cm in length, transarterial chemoembolization is suggested to shrink tumors first (18). These results suggest that neoadjuvant chemoradiotherapy combined with liver transplantation is a new treatment option for advanced ICCA. In addition to OLT, neoadjuvant chemoradiotherapy can also allow patients with initially unresectable locally advanced cholangiocarcinoma to be reclassified as surgical candidates in a substantial proportion. A study conducted by Sumiyoshi et al. found that conventional radiotherapy with a dose of 50 Gy/25F combined with chemotherapy such as S1 or CPT11 for advanced ICCA resulted in a partial response (PR) rate of 57.1%, a radical tumor resection rate of 71%, and a postoperative median DFS of 21.5 months (19). Given this, the CSCO guidelines suggest that neoadjuvant radiotherapy and chemotherapy should be considered for ICCA under the following conditions: intrahepatic lesion ≤ 6 cm, intrahepatic lesion, and lymph node metastasis are within the surgical resection range. In such cases, a conventional radiotherapy dose of 45–50.4 Gy/25–28F or SBRT dose of 40 Gy/5F can be administered; fluorouracil is the main drug in concurrent chemotherapy (14).

The history of neoadjuvant therapy for ECCA dated back to 20 years ago. As early as 1983–1996, the MD Anderson Center conducted a retrospective analysis on the treatment of advanced CCA and found that 9 cases of advanced ECCA were treated with neoadjuvant radiotherapy and chemotherapy, and the radiotherapy dose was 45–50.4 Gy/25–28F combined with 5-FU chemotherapy. The results showed that the R0 resection rate of these 9 patients reached 100%. Pathologic complete response was achieved in 3 cases, suggesting for the first time that neoadjuvant radiotherapy is feasible for advanced ECCA (20). In 2015, Kobayashi et al. conducted a phase I clinical trial of gemcitabine-based neoadjuvant concurrent chemoradiotherapy in patients with advanced ECCA. Twenty-five patients with inoperable advanced ECCA were enrolled in this study. After neoadjuvant chemoradiotherapy, the R0 resection rate was 96%, the 3-year survival rate was 74.6%, and the toxicities associated with treatment were low. This study further confirms the efficacy and safety of neoadjuvant chemoradiotherapy in advanced CCA (21). In a retrospective analysis, Jung et al. and Kobayashi et al. respectively enrolled 57 and 106 ECCA patients with the stage above T3, or with vascular invasion or lymph node metastasis, and administered gemcitabine combined with conventional radiotherapy and compared it with surgery alone. The results showed that neoadjuvant chemoradiotherapy not only effectively achieved preoperative downgrading but also substantially increased DFS and OS (22, 23). Therefore, the CSCO guidelines suggest that preoperative radiotherapy and chemotherapy can be considered for locally advanced ECCA with T3 or higher stages and positive lymph nodes. Based on existing studies, conventional radiotherapy with a dose of 40-45 Gy (1.8–2.0 Gy/F) is recommended as the main treatment, while concurrent chemotherapy drugs recommended are fluorouracil and gemcitabine; further, attention should be paid to relevant toxicity (14).

### Palliative Radiotherapy for Advanced CCA

Although systemic therapy is the preferred treatment for advanced CCA, the prognosis is usually poor, with a median survival of 3 to 9 months (24). Existing studies have shown that symptom-based palliative radiotherapy can alleviate local symptoms and improve local control rates for locally advanced CCA, thereby improving the quality of life of patients with advanced CCA.

Conventional fraction radiotherapy combined with concurrent chemotherapy is a widely accepted palliative therapy. A total of 84 patients with inoperable ICCA were included in the retrospective study conducted by Chen et al. in 2010; 35 of them received external irradiation (dose range of 30–60 Gy; median dose 50 Gy; 1.8–2.0 Gy/F), the median survival (9.5 months vs. 5.1 months, P=0.003) and 1-year survival rate (38.5% vs. 16.4%) were significantly improved in the radiotherapy group compared with the non-radiotherapy group (25). A phase II single-arm study in 2016 included 27 patients with locally advanced ECCA who received gemcitabine combined with cisplatin synchronous radiotherapy (50 Gy). The median follow-up time was 16 months, and the results showed that the 2-year local control rate was 29%, and the 2-year and 3-year OS were 27% and 7%, respectively (26). A prospective exploratory study in South Korea in 2016 enrolled 18 patients with locally advanced CCA who received gemcitabine and cisplatin combined with radiotherapy (45 Gy/25F), and the results showed that the objective response rate of these patients was 27.8%, the median PFS and median OS were 6.8 and 9.6 months, respectively. The result showed that fluorouracil and gemcitabine-based concurrent chemoradiotherapy had a survival benefit in patients with unresectable and metastatic biliary malignancy (27).

Concurrently, existing studies have shown that SBRT has advantages over conventional radiotherapy. In a phase I clinical trial of SBRT conducted by Tse et al. in 2008 for unresectable ICCA patients, the median dose was 32.5 Gy (28.2–48 Gy, 4-9Gy/F), the median survival time was 15.0 months, and the 1-year OS reached 58%, which was significantly improved compared with conventional radiotherapy (28). In a study by Polistina et al. in 2011, the median survival time of hilar CCA patients treated with SBRT (30 Gy/3F) combined with gemcitabine was 35.5 months, and the 2-year OS was 80%, which was much higher than that of patients treated with conventional radiotherapy and chemotherapy alone (29). In 2019, Lee et al. screened 11 studies for the treatment of unresectable advanced CCA, including ECCA and ICCA patients, and the results showed that the overall one-year local control rate was approximately 78.6%, with low radiation-related toxicity (30). Existing studies have shown that the therapeutic efficacy of SBRT is closely related to the biologic equivalent dose (BED) of radiotherapy. A retrospective analysis conducted by Tao et al. in 2016 for unresectable ICCA included 79 patients with a median dose of 58.05 Gy (35–100 Gy/3–30F); the results showed that the high-BED group (> 80.5 Gy) exhibited improved 2-year OS rate (73% vs. 58%) compared to the low-dose group (≤ 80.5 Gy), and confirmed the survival benefit in unresectable CCA patients was associated with dose escalation (31).

**Figure 1:**
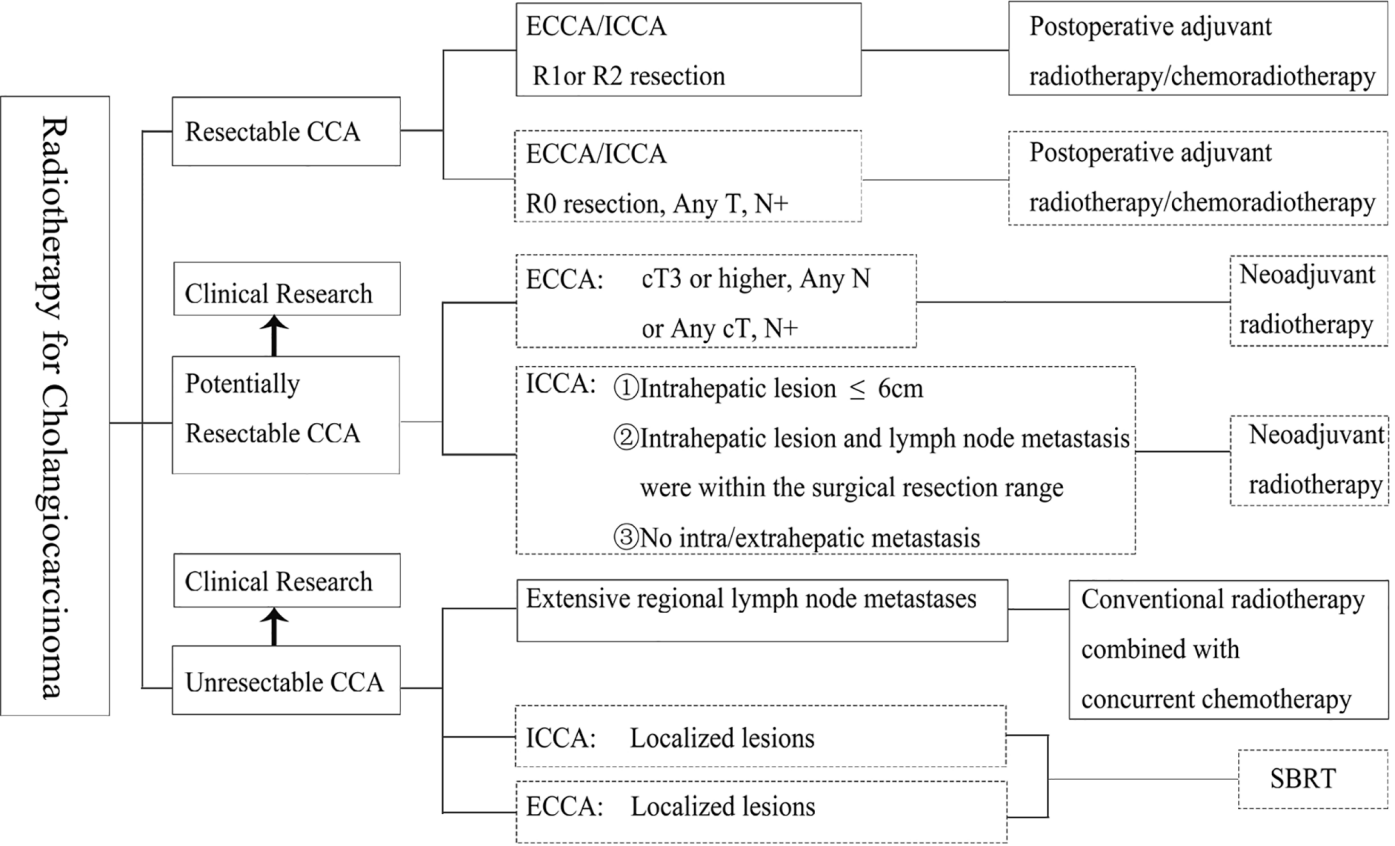
Principles of radiation in different cholangiocarcinoma stages. ECCA, extrahepatic cholangiocarcinoma; ICCA, intrahepatic cholangiocarcinoma; R0, no cancer at resection margins; R1, microscopic residual cancer; R2, macroscopic residual cancer; SBRT, stereotactic body radiation therapy. The boxes with solid lines indicate Category I or II recommendations and the boxes with dotted lines indicate Category III recommendations.

Based on the appeal studies, the CSCO guidelines recommend that in patients with unresectable CCA, priority be given to conventional-dose radiotherapy combined with concurrent chemotherapy, especially when extensive lymph node metastasis is present and the radiotherapy target area is large. Patients with localized CCA should be treated with SBRT. The radiation dose in the tumor area and lymphatic drainage area is 45–50.4 Gy, and 1.8–2.0 Gy for a single time. According to patient tolerance, the dose in the tumor area can be increased to 60 Gy or higher, and the dose for organs at risk should be considered in the treatment. For high-dose and low-fraction-radiation therapy such as SBRT, it is recommended to irradiate only the primary tumor and metastatic lymph nodes, and not to include high-risk lymph node drainage areas. At present, there is no unified dose model for SBRT as a standard recommendation, and the dose segmentation can be referred to like 30–50 Gy/3–5F. The determination of single segmentation dose and segmentation time depends on the distance between the target area and organs at risk and the number of organs at risk (14). The current recommendations for radiotherapy for CCA are summarized (**Figure 1**).

## Timing of Radiotherapy

Currently, there is a lack of many prospective studies to guide the decision regarding radiotherapy planning. The timing of radiotherapy depends on the disease status of CCA patients. For patients with resectable CCA, adjuvant radiotherapy should be considered after surgical treatment, and the optimal time to start postoperative adjuvant radiotherapy has not been determined. In SWOG S0809, the phase II clinical trial in 2015, postoperative patients with CCA were treated with gemcitabine combined with capecitabine (biweekly regimen) for 4 cycles, and patients without progression were administered capecitabine combined with radiotherapy (regional lymph node 45 Gy, preoperative tumor bed 54–59.4 Gy), which was widely referenced (15). Among other retrospective studies, in a study on adjuvant radiotherapy for ECCA in 2000, 34 patients were treated with intraoperative radiotherapy, and in 22 of them, external radiotherapy was initiated 4–6 weeks postoperatively (32). In a retrospective study in 2007 that included 34 patients, the median time to start chemoradiotherapy was 53 days (43–62 days) after surgery (33). Therefore, considering the physical recovery of postoperative patients and the results of the current prospective phase II clinical study SWOG S0809 (15) as well as the existing retrospective study results, it can be considered that adjuvant concurrent chemoradiotherapy should be started 8 weeks after surgery. If combined with postoperative adjuvant chemotherapy, 2 to 4 cycles of postoperative adjuvant chemotherapy can be performed first, followed by concurrent chemoradiotherapy (14).

The timing of neoadjuvant radiotherapy for CCA patients with potential resection is inconclusive. It is generally considered to perform the downgrading after neoadjuvant radiotherapy to reevaluate the possibility of surgery, so it needs to be carried out after a multidisciplinary team (MDT) discussion. For patients with unresectable and metastatic CCA, the existing evidence has shown that palliative radiotherapy can improve the quality of life and local control rate in both ICCA and ECCA (31, 34). However, there is no clear definition of the time when palliative radiotherapy should be involved. Based on the existing research, it is recommended that for patients with advanced cholangiocarcinoma, under the condition of acceptable physical condition, palliative radiotherapy is feasible for the lesions with distant organ metastasis, such as liver, lung, bone, and retroperitoneum, when surgery or intervention are impossible, to relieve symptoms and improve local control. The radiotherapy mode (intensity-modulated conformal radiotherapy or SBRT) and the timing of radiotherapy intervention can be implemented with the participation of the MDT (14).

## Target Area Determination

CCA radiotherapy requires accurate definition and delineation of target areas. Accurate delineation of tumor volume can achieve a higher local control rate and reduce radiation-related toxicity. The clinical target volume (CTV) is usually determined based on imaging techniques such as CT or MRI to determine visible tumors. In a 2021 study comparing CT, MR, and PET/MR for CCA target delineation, the gross target volume (GTV) delineated on PET/MR was significantly larger than the target volume delineated on CT and MR, and there was no significant difference between the target delineated on CT and MR. This study showed that compared with CT or MR, targeting CCA based on 18F-FDG PET/MR enables more accurate detection of positive lymph nodes, reducing the risk of missing lymph nodes, and thus accurately defining the GTV (35). However, a unified definition of the target description of CCA is still lacking.

**Figure 2:**
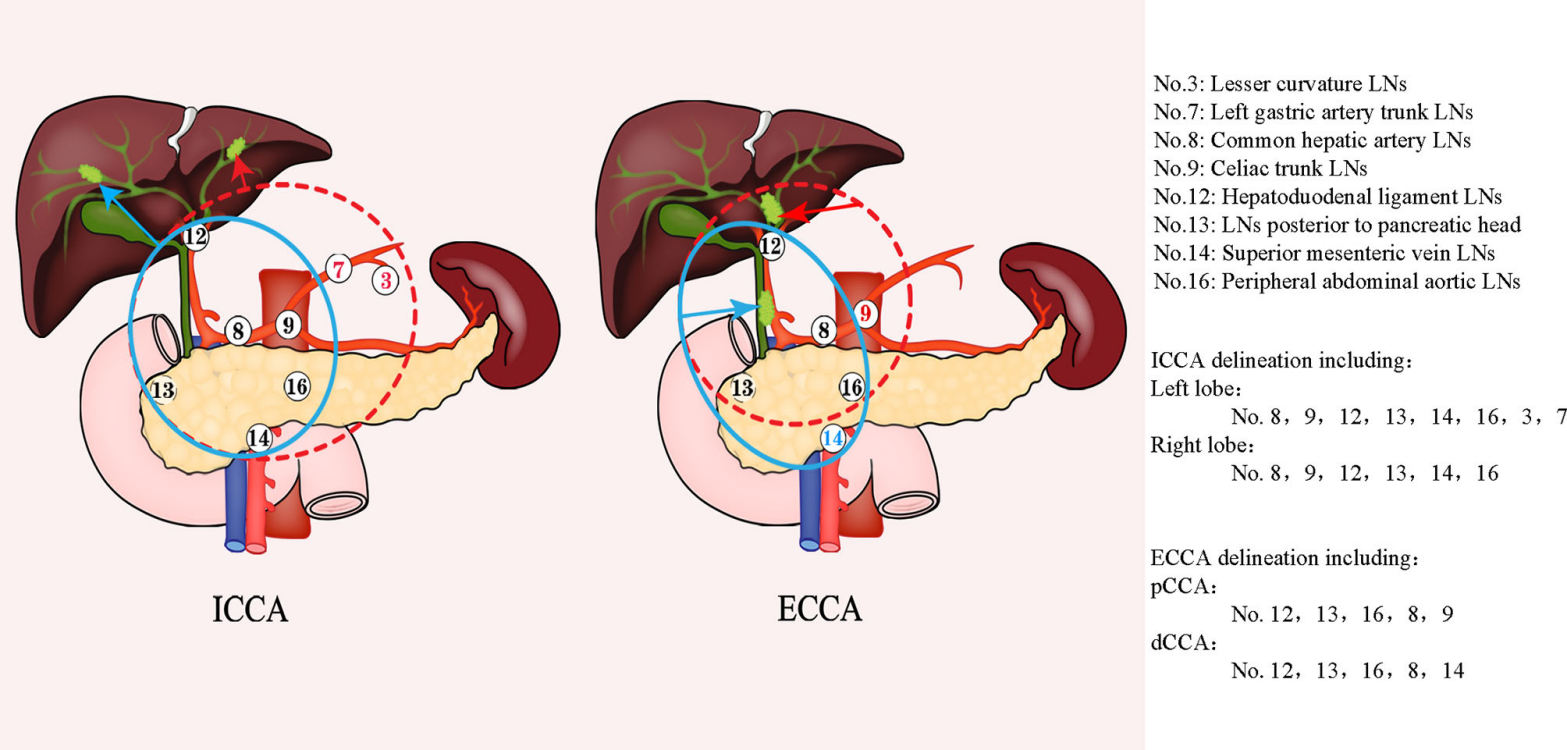
Lymph node delineation in cholangiocarcinoma. The left figure shows the range of LNS with high risk in ICCA, the red dotted line represents the delineation range of LNS for left ICCA, and the blue line represents corresponding LNS for right ICCA; The right figure represents the corresponding the range of LNS with high risk for ECCA, the red dotted line represents the corresponding LNS area for pCCA, and the blue line represents the corresponding lymph node drainage area of dCCA. ECCA, extrahepatic cholangiocarcinoma; ICCA, intrahepatic cholangiocarcinoma; pCCA, perihilar cholangiocarcinoma; dCCA, distal cholangiocarcinoma; LNS, lymph node stations.

In 2017, Socha et al. further defined the lymph node region of CTV in radiotherapy planning by comparing the postoperative recurrence of the existing research data. In the implementation of past radiotherapy plans, some lymph nodes may be potentially missed, and in ECCA and gallbladder cancer, unnecessary lymph nodes are also included in the target area. therefore, it is necessary to determine the radiotherapy range of high-risk lymph nodes according to the location of the primary tumor (34). In 2017, Marinelli et al. Observed and studied the correlation between the location of primary biliary tract tumor and lymph node involvement rate. They found that for patients with ICCA, the drainage area of high-risk lymph nodes should vary according to the location of primary focus, while for patients with ECCA, the target area should include primary tumor bed and regional lymph nodes, it should be noted that hilar tumors still need to include liver margin and anastomosis (36). Based on the data, the CSCO guidelines recommend that the lymphatic drainage areas be classified according to the tumor site as follows: for ICCA, high-risk lymph node drainage area should include the hilar lymph node, hepatoduodenal lymph node, celiac trunk lymph node, posterior pancreatic head lymph node, mesenteric lymph node, and para-aortic lymph node drainage area. If the primary focal point of ICCA is in the left hepatic lobe, the high-risk lymph node drainage area should include the lesser curvature of the stomach and left gastric lymph node drainage area. For pCCA, it should include the hepatoduodenal lymph nodes, hilar lymph nodes, celiac trunk, epigastric para-aortic lymph nodes and lymph nodes behind the head of the pancreas. While for dCCA, it should include hilar lymph nodes, hepatoduodenal lymph nodes, retro pancreatic lymph nodes, mesenteric lymph nodes, and the abdominal aortic drainage area. For the celiac trunk lymph nodes, their inclusion should be considered based on the imaging evaluation results, considering their low recurrence rate (14, 34, 36). Therefore, the lymph node drainage area at high risk of CCA is shown in **Figure 2**.

ICCA usually presents on CT as an unencapsulated homogeneous mass with irregular margins, low density, and irregular peripheral enhancement (37, 38). In 2002, Ebata et al. found that 80 of 253 cases had a microscopically positive margin with a median diffusion distance of 10 mm (39). Similarly, in the 2009 study of Bi et al, analyzed the preoperative imaging and postoperative pathological evaluation and found that the scope of the lesion under the microscope in the pathological evaluation was approximately 0.4–8.0 mm larger than that in imaging (40). Therefore, in the case of conventional radiotherapy, it is suggested that the radiotherapy target area of biliary malignancy should include any tumor area seen in imaging; CTV should be expanded 10 mm based on GTV, the postoperative tumor bed should be included, and anastomosis should be included when the postoperative resection margin is positive. While considering the respiratory mobility and positioning error, PTV should be expanded 5 mm and up and down 7 mm based on CTV. It should be noted that, for high-dose and low-segmentation radiotherapy such as SBRT, it is recommended to irradiate only the primary tumor and metastatic lymph nodes, and not to include the high-risk lymph node drainage areas (14).

## Latest Progress in Radiotherapy

### Proton Radiotherapy

Proton radiotherapy can be used as a new technique in the treatment of CCA, thereby protecting normal liver parenchyma and other adjacent organs due to its rapid energy absorption and effective dose reduction (41, 42). Several retrospective and prospective studies have demonstrated superior efficacy of proton therapy in the treatment of cholangiocarcinoma and relevant studies are summarized in **Table 1** (43–47). The highest level of evidence to date is a phase II study of high-dose fractionated proton beam therapy for advanced ICCA and HCC in 83 assessable patients, including 37 patients with CCA. The median dose was 58.0 Gray equivalent (GyE). The results showed that the 2-year local control rate of ICCA was 94.1% and the 2-year survival rate was approximately 46.5%, and the incidence of grade 3 or higher radiation-related adverse reactions was only 7.7% in patients with ICCA (45). In 2020, Hung et al. conducted a retrospective analysis of the data of 30 patients with CCA who received proton therapy from 2015 to 2017. The 1-year local control rate of the 30 patients was 88%, and the median PFS was 10.4 months. Among them, 23 patients who received synchronous chemotherapy had a greater survival benefit, and the median PFS was 12.1 months. Meanwhile, the regimen was well tolerated, and the incidence of grade 3 or above adverse reactions was approximately 7% (47). From 2015 to 2017, a prospective study included 30 patients with unresectable CCA, who received large-fraction proton beam therapy with a median dose of 72.6 GyE; the 1-year local control rate was 88%, and 1-year OS was 83% (48). Therefore, compared with conventional radiotherapy, proton therapy has exhibited the advantages of safety and survival benefit.

**Table 1:** Studies in Proton Therapy for Cholangiocarcinoma.

Study	Type	Enrollment	Total dose (Gy)	mFollow up (months)	LCR	PFS	OS	Toxicity
Makita et al. (43)	R	28	68.2Gy (RBE)	12	BED > 70Gy:	mPFS=8.0m	mOS=12.0m	≥grade 2 acute treatment-related toxicities=8
					1y-LCR=83.1%	1y-PFS=29.5%	1y-OS=49.0%	≥grade 2 late gastrointestinal toxicities=7
					BED ≤ 70Gy:			
					1y-LCR=22.2%			
Ohkawa et al. (44)	R	20	intrahepatic region=	19.3	Curative group:	n.r	Curative group: mOS=27.5m	≥grade 3 acute treatment-related toxicitie=1
			72.6GyE/22F		1y-LCR=88%		1y-OS=82%	grade 3 biliary tract infection=2
			lymph node=		3y-LCR=60%		3y-OS=38%	
			56.1GyE/17F				Palliative group:	
							mOS=9.6m	
							1y-OS=50%	
							3y-OS=0%	
Hong et al. (45)	P	39	67.5GyE/15F	19.5	2y-LCR=94.1%	mPFS=8.4m	mOS=22.5m	fatigue=65.1%
						1y-PFS=41.4%	1y-OS=69.7%	rash=61.4%
						2y-PFS=25.7%	2y-OS=46.5%	nausea=30.1%
								grade 3 radiation-related toxicities=7.7%
Shimizu et al. (46)	R	37	72.6GyE/22F	37.5	Curative group:	Curative group:	Curative group:	grade 3 biliary tract infections=3
					1y-LCR=100%	mPFS=19.0m	mOS=25m	
					2y-LCR=71.5%	1y-PFS=58.5%	1y-OS=66.3%	
						2y-PFS=37.6%	2y-OS=52.4%	
							Palliative group:	
							mOS=7m	
							1y-OS=50%	
							3y-OS=0%	
Hung et al. (47)	R	30	72.6GyE	16	1y-LCR=88%	mPFS=10.4	mOS=19.3m	grade 3-4 toxicities=3
						1y-PFS=47%	1y-OS=83%	radiation-induced liver disease=2
							2y-OS=32%	

n.r, not reported; R, retrospective study; P, prospective study; CCA, cholangiocarcinoma; LCR, local control rate; OS, overall survival; PFS, progress free survival; 1y-PFS, 1-year progress free survival rate; 2y-PFS, 2-year progress free survival rate; 1y-LCR, 1-year local control rate; 2y-LCR, 2-year local control rate; 3y-LCR, 3-year local control rate; 1y-OS, 1-year overall survival; 2y-OS, 2-year overall survival; 3y-OS, 3-year overall survival; GyE,Gray equivalents;RBE,relative biological effectiveness.

### Targeted Therapy and Immunotherapy Combined With Radiotherapy

The development of precision medicine also brings more treatment opportunities for patients with bile duct cancer (49). New drug options are available for advanced CCA, such as the combination of dabrafenib and trametinib has produced promising results for BRAFV^600E^-mutated CCA (50), and isocitrate dehydrogenase (IDH1) inhibitor has also revealed successful results for biliary tumors (51, 52).

In addition to this, current immunotherapy represented by immune checkpoint inhibitors has shown significant advantages in a variety of malignancies (53). Radiation therapy has a direct cytotoxic effect on tumor cells and can produce certain anti-tumor immune responses by influencing the microenvironment and affecting distant tumor cells by releasing proinflammatory cytokines and chemokines to mobilize systemic immune cells (54). The immunomodulatory effects of radiotherapy have been widely reported in pre-clinical and clinical studies. Radiotherapy releases tumor antigens and facilitates the regulation of immune pathways, increasing tumor antigen presentation, initiating tumor-specific cytotoxic T cells, and enhancing T cell homing. This distant effect can be further enhanced by a combination of radiotherapy and immunization regimens (55). There is preclinical and clinical evidence to indicate that SBRT in combination with immunotherapy is more likely to activate the immune response in the tumor area than conventional radiotherapy (56). In a case report in 2020, a stage IV ICCA patient received radiotherapy combined with 6 cycles of immunotherapy with PD-1 receptor inhibitors to treat lung metastases and intrahepatic lesions. The efficacy of the treatment in the patient was assessed as complete response (CR), and no significant treatment-related adverse reactions were observed. The survival time after combined therapy exceeded 26 months (57). Subsequently, Zhao et al. reported four cases of SBRT combined with immune checkpoint inhibitors in the treatment of ICCA or hilar CCA in 2021, respectively achieving CR, PR, and stable disease, and even one patient who was initially inoperable received surgery (58). Although there is little evidence at present, immunotherapy combined with radiotherapy shows a good prospect, which needs to be further explored. Infiltrating the immune cell distribution and type of bile duct tumor at the same time also has a certain relationship with the prognosis. In EHCC, infiltration of CD4^+^, CD8^+^ cytotoxic T lymphocytes, and B lymphocyte/plasma cells could achieve a favorable prognosis, while the increase of macrophages would herald an advanced disease (59). Studies have shown that changes in tumor tissue immune status after radiotherapy are related to therapeutic effect, and patients with persistently high levels of PD-L1 and CD8^+^ tumor-infiltrating lymphocyte expression in tumor tissues before and after CRT have a poor prognosis (60).

Therefore, the application of immune checkpoint inhibitors or targeted drugs combined with radiotherapy in CCA patients is worthwhile. The relevant clinical studies are summarized in **Table 2**. Represent studies are the phase II clinical trial of PD-1 inhibitor combined with radiotherapy in the treatment of advanced ICCA in China (NCT03898895), the phase I–II clinical trial of peposertib plus avelumab combined with radiotherapy in the treatment of advanced solid tumors, and hepatobiliary malignancies in the United States (NCT04068194), and the phase I clinical trial (NCT04708067) of bintrafusp alfa combined with high-dose fractionated radiotherapy in the treatment of advanced ICCA. The results of these studies could provide valuable information on such combined therapies.

**Table 2:** Clinical Studies of Immunotherapy or Targeted Therapy Combined with Radiotherapy for cholangiocarcinoma.

Trail ID	Title	Status	Population	Study Arms	Phase	Initiation Date
NCT03898895	Combination of Radiotherapy with Anti-PD-1 Antibody for Unresectable Intrahepatic Cholangiocarcinoma	Recruiting	ICCA	Radiotherapy+anti-PD-1 vs. Gemcitabine + cisplatin	Phase 2	December 1, 2019
NCT04708067	Hypofractionated Radiation Therapy and Bintrafusp Alfa for the Treatment of Advanced Intrahepatic Cholangiocarcinoma	Recruiting	Locally Advanced ICCA	Single-arm: Bintrafusp Alfa + Biopsy + Hypofractionated Radiation Therapy	Phase 1	August 31, 2021
			Metastatic ICCA			
NCT04648319	A Study of BMS-936558 With SBRT After Induction Chemotherapy in Cholangiocarcinoma	Recruiting	CCA	Single-arm: BMS-936558+SBRT	Phase 2	April 15, 2021
NCT00426829	Proton Therapy and Bevacizumab for PrimaryLiver Tumors	Terminated	Liver Cancer	Single-arm: Bevacizumab+ Proton Radiation Therapy	Phase 1	May 2007
			Hepatocellular Carcinoma			
			CCA			
NCT04068194	Testing the Combination of New Anti-cancer Drug Peposertib with Avelumab and Radiation Therapy for Advanced/Metastatic Solid Tumors and Hepatobiliary Malignancies	Recruiting	Locally Advanced CCA	Avelumab+Hypofractionated Radiation Therapy vs. Hypofractionated Radiation + Avelumab + Peposertib	Phase 1	December 23, 2019
			Locally Advanced GBC		Phase 2	
			Locally Advanced Malignant Solid Neoplasm			
			Metastatic CCA			
NCT00266097	Oxaliplatin, Gemcitabine, Erlotinib, and Radiation Therapy in Treating Patients with Unresectable and/or Metastatic Pancreatic Cancer or Biliary Tract Cancer	Completed	ECCA	Oxaliplatin + Gemcitabine + Radiation vs. Erlotinib + Oxaliplatin + Gemcitabine + Radiation	Phase 1	August 2004
			GBC			
			Pancreatic Cancer			

CCA, cholangiocarcinoma; ICCA, intrahepatic cholangiocarcinoma; ECCA, extrahepatic cholangiocarcinoma; GBC, gallbladder cancer; PD-1: programmed cell death 1; SBRT, stereotactic body radiotherapy.

## Conclusion

The European Society of Medical Oncology (ESMO), National Comprehensive Cancer Network (NCCN), and American Society of Clinical Oncology (ASCO) guidelines for hepatobiliary malignancies are still being updated, and the CSCO guidelines for biliary tract cancer have also been launched and updated in recent years. Drug therapy is the mainstay of treatment for advanced cholangiocarcinoma, radiation therapy is still necessary for some patients. The radiotherapy in the existing NCCN, ESMO, and ASCO guidelines is relatively few recommend and lacks a detailed description of radiotherapy mode, dose, and target area. The CSCO guidelines published the first edition of the diagnosis and treatment guidelines for biliary tract cancer in 2020 and elaborated on the relevant content of radiotherapy in detail. In the past two decades, there have been advances in radiotherapy and systemic therapy for CCA, with the survival benefit of postoperative adjuvant radiotherapy for CCA being recognized, and more evidence of SBRT therapy for patients with advanced CCA. The high local recurrence rate in CCA patients with R1 resections provides a basis for postoperative adjuvant radiotherapy. At present, there are many clinical projects under development for CCA radiotherapies, such as a phase III clinical trial in India (NCT02773485) comparing chemotherapy alone with chemotherapy combined with high-dose radiotherapy in the treatment of unresectable CCA, a prospective multicenter study of liver transplantation after neoadjuvant radiotherapy and chemotherapy for unresectable hilar CCA in Spain (NCT04378023), and a phase II trial of SBRT combined with chemotherapy for CCA in Belgium (NCT04648319). It is believed that these clinical projects can provide strong evidence for radiation therapy for CCA.

Nowadays, advances in imaging technology and radiotherapy technology provide prospects for the implementation of precise radiotherapy. In the future, based on tumor biological characteristics, biomarkers, and the direction of tumor microenvironment combined with appropriate imaging and radiotherapy, developing technologies will become the trend in individual precision radiotherapy, further reducing the toxic and side effects of radiotherapy and improving the prognosis of patients with CCA.

## Author Contributions

HM, YoX and AH contributed to the conception and design of the study. YuX organized the database. NW wrote the first draft of the manuscript. BK, AH, HM, and NW wrote sections of the manuscript. All authors contributed to manuscript revision, read, and approved the submitted version.

## Funding

This study was supported by the Chinese Society of Clinical Oncology (CSCO) (Y-SY201901-0014).

## Conflict of Interest

The authors declare that the research was conducted in the absence of any commercial or financial relationships that could be construed as a potential conflict of interest.

## Publisher’s Note

All claims expressed in this article are solely those of the authors and do not necessarily represent those of their affiliated organizations, or those of the publisher, the editors and the reviewers. Any product that may be evaluated in this article, or claim that may be made by its manufacturer, is not guaranteed or endorsed by the publisher.
